# Surgical Notes

**Published:** 1859-11

**Authors:** 


### SURGICAL NOTES.

1. *Vesica-Vaginal Fistula.*

This operation was performed on the 24th Sept., with the assistance of Profs. Miller and Ingalls and Dr. Durham, on a woman, 24 years of age, delivered of her first child by instruments, above four months previously. The fistula was large enough to admit the finger into the bladder. The vagina was drawn into bands and contracted in different directions, particularly at its orifice.

The operation was performed in the manner used at present. We have been in the habit of using the lead wire for patients long before Dr. Sims performed his operation, and as it always succeeded in favorable cases, we were not disposed to abandon it.

However, in this case we used the silver wire, and found it to answer in every respect as well as the lead, and in one important particular, better. It remains longer in the tissues without producing ulceration. Five stitches were used, the needle being introduced by the instrument of Matthieu, instrument maker of Paris, (whether invented by him or not we are unable to say.) Although the catheter left in the bladder was once obstructed, and once drawn out by accident, the operation succeeded perfectly, and three weeks after the operation the opening was closed, but the sphincter did not resume its function. There was also paralysis of the left foot, which dates from the time of her delivery.

2. *Resection of the Knee Joint.*

In the No. of this Journal for July, 1858, we reported a case of this operation on a boy for caries. In the No. for June, 1858, the sequel and ultimate result of that case are given. It was entirely successful in preserving a useful member.

About the first of March last, we performed the operation on a boy about twelve years old, for caries. This was an unfavorable case on account of the extent of the disease, and from the boy having had previously a fracture of the femur of the side affected, which had united with considerable shortening. We propose to give the result of this case at some future time, when the usefulness of the member shall have been fully ascertained. We have already referred to it in a note to the discussion of the Society of Surgery of Paris, on the subject. At present we are able to state that the boy is in good health, that full bony union and in good position has taken place, and there only remain these small fistulous openings, through which a few drops of pus are discharged daily.

On the 5th of October, 1859, we performed resection of the knee for ulceration of the cartilages and disease of the bone, at the City Hospital, in presence of the medical class. Two and a half inches of the femur and the articular surfaces of the tibia were removed. This patient was much reduced, and after the operation was greatly depressed, but reacted the third day. At present he is doing well, the wound of skin is healed, except at one point; he suffers no pain, and there is but a small discharge of pus, which is healthy.

3. *Congenital Absence of the Vagina, with imperfect development of the Uterus.*

Since the report of cases of the No. of this Journal for July, 1859, we have been consulted in behalf of three patients affected with this congenital defect.

On one of these the operation was performed, Sept. 24. Professors Miller and Ingalls assisted. The patient was twenty years of age, and had been married three years.

There was full development of the breasts and external organs of generation. Pain occurred at regular monthly intervals.

By examination *per rectum*, a body was felt in the situation of the uterus, having the characters of that organ when imperfectly developed. The vagina was closed at the orifice and throughout its whole length. The operation was performed in the manner described in the article referred to. No difficulty was found in forming a passage sufficiently large to receive a large sized oval pessary without opening the bladder, rectum or peritonæum.

No dangerous effects followed. The final result cannot as yet be judged of, but will be reported when sufficient time shall have elapsed to enable a correct judgment to be formed. In the meantime appearances thus far confirm us in the opinion heretofore expressed in favor of this operation.

